# Gelatin‐polysaccharide composite scaffolds for 3D cell culture and tissue engineering: Towards natural therapeutics

**DOI:** 10.1002/btm2.10124

**Published:** 2018-12-28

**Authors:** Samson Afewerki, Amir Sheikhi, Soundarapandian Kannan, Samad Ahadian, Ali Khademhosseini

**Affiliations:** ^1^ Biomaterials Innovation Research Center, Division of Biomedical Engineering, Dept. of Medicine Brigham and Women's Hospital, Harvard Medical School Cambridge MA 02142; ^2^ Harvard‐MIT Division of Health Sciences and Technology Massachusetts Institute of Technology Cambridge MA 02139; ^3^ Center for Minimally Invasive Therapeutics (C‐MIT) University of California‐Los Angeles Los Angeles CA 90095; ^4^ California NanoSystems Institute (CNSI) University of California‐Los Angeles Los Angeles CA 90095; ^5^ Dept. of Bioengineering University of California‐Los Angeles Los Angeles CA 90095; ^6^ Nanomedicine Division, Dept. of Zoology Periyar University Salem Tamil Nadu India; ^7^ Dept. of Radiological Sciences, David Geffen School of Medicine University of California‐Los Angeles Los Angeles CA 90095; ^8^ Dept. of Chemical and Biomolecular Engineering University of California‐Los Angeles Los Angeles CA 90095; ^9^ Dept. of Bioindustrial Technologies, College of Animal Bioscience and Technology Konkuk University Seoul Republic of Korea

**Keywords:** 3D cell culture, gelatin, polysaccharides, scaffold, therapeutics, tissue engineering

## Abstract

Gelatin is a promising material as scaffold with therapeutic and regenerative characteristics due to its chemical similarities to the extracellular matrix (ECM) in the native tissues, biocompatibility, biodegradability, low antigenicity, cost‐effectiveness, abundance, and accessible functional groups that allow facile chemical modifications with other biomaterials or biomolecules. Despite the advantages of gelatin, poor mechanical properties, sensitivity to enzymatic degradation, high viscosity, and reduced solubility in concentrated aqueous media have limited its applications and encouraged the development of gelatin‐based composite hydrogels. The drawbacks of gelatin may be surmounted by synergistically combining it with a wide range of polysaccharides. The addition of polysaccharides to gelatin is advantageous in mimicking the ECM, which largely contains proteoglycans or glycoproteins. Moreover, gelatin–polysaccharide biomaterials benefit from mechanical resilience, high stability, low thermal expansion, improved hydrophilicity, biocompatibility, antimicrobial and anti‐inflammatory properties, and wound healing potential. Here, we discuss how combining gelatin and polysaccharides provides a promising approach for developing superior therapeutic biomaterials. We review gelatin–polysaccharides scaffolds and their applications in cell culture and tissue engineering, providing an outlook for the future of this family of biomaterials as advanced natural therapeutics.

## 1 INTRODUCTION

Biomaterials play a pivotal role in designing functional scaffolds, providing three‐dimensional (3D) templates that facilitate cell adhesion, growth, proliferation, and differentiation. Engineered scaffolds may promote vascularization and tissue formation, which are essential for tissue engineering and regenerative medicine.^1^ Biomaterials may be prepared from natural polymers,^2^ such as alginate, gelatin, chitosan, hyaluronic acid (HA), and collagen, or be made up of synthetic polymers,^3^ such as poly(ethylene glycol) (PEG), poly‐l‐lactic acid (PLLA), polycaprolactone (PCL), and poly(lactic acid‐co‐caprolactone). One of the major challenges in designing biomaterial scaffolds is to modify their building blocks to mimic the extracellular matrix (ECM) in the native tissues. ECMs consist of an acellular 3D network of various amino acid‐ and sugar‐based macromolecules, which bring cells together, support them, and control tissue structures. Simultaneously, they regulate the cell function and morphogenesis and facilitate the diffusion of nutrients, metabolites, and growth factors.^4^ In this context, hydrogels have played a crucial role by providing structural similarities to the biomacromolecules found in the ECM, leveraging cellular functions and enhancing the permeability of oxygen, nutrients, and other water‐soluble metabolites.^5^, ^6^ Hydrophilic polymeric networks in hydrogels can take up and maintain liquids (swell) when exposed to an aqueous medium. These properties render hydrogels an attractive class of biomaterials for 3D cell culture.^7^, ^8^, ^9^, ^10^, ^11^

Gelatin is one of the most common biomaterials for 3D cell culture, providing suitable chemical and biological cues for hosting a variety of cells. Despite a broad spectrum of applications, poor mechanical properties, fast enzymatic degradation, and low solubility in concentrated aqueous media are among the limitations of gelatin.^12^, ^13^ To overcome these shortcomings, gelatin has been combined with polysaccharides. Compared to synthetic polymers, such as PEG,^14^ PCL,^15^ poly(lactic‐co‐glycolic acid) (PLGA),^16^ and PLLA,^17^ polysaccharides–gelatin composite biomaterials better resemble the native ECM. The integration of gelatin and polysaccharides not only resembles the glycoproteins in the ECM but also introduces new synergistic characteristics that would otherwise be impossible to achieve using solely one of the materials. This strategy may serve as a powerful tool for designing complex hybrid polymeric frameworks in a broad spectrum of tissue engineering applications.

To mimic the key physiological features of ECM, proper peptides/proteins, cell‐signaling factors, enzyme‐sensitive moieties, and growth factors must be conjugated to polysaccharides. Cell behavior can be directed to develop functional tissues via engineering gelatin–polysaccharide hybrid 3D scaffolds using chemical, physical, and mechanical modifications.^18^ For instance, conjugating integrin, selectin, and CD44 to polysaccharides have imparted cell‐adhesive domains to the hybrid scaffolds, supporting cell functions and organization.^19^, ^20^ Moreover, since both components are green materials derived from natural resources, they also contribute to eco‐technology and sustainable material design.

**Figure 1:**
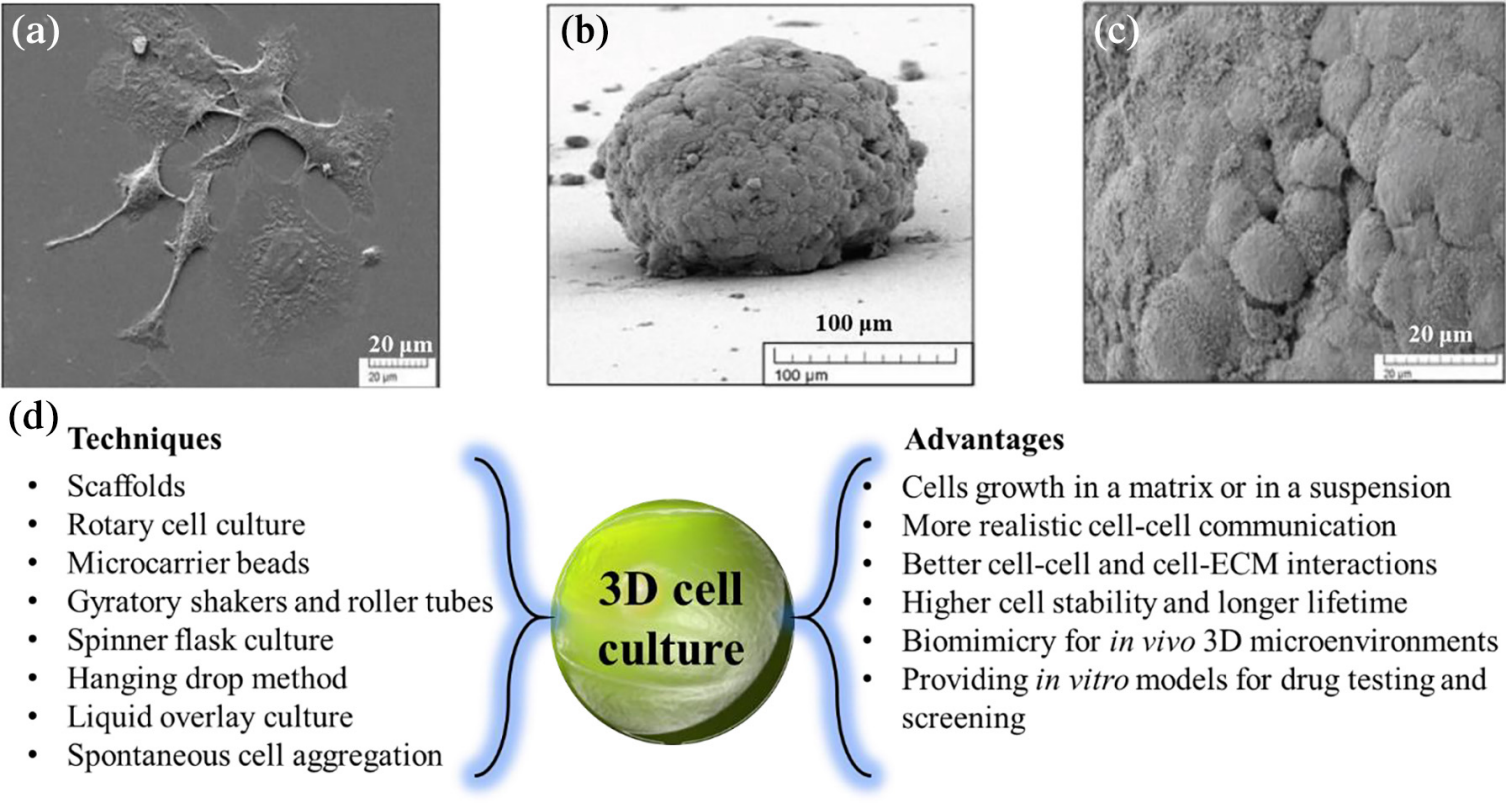
(a) Scanning electron microscopy (SEM) micrographs of HER2‐overexpressing cell lines (HCC1954) in 2D (scale bar is 20 μm) and (b) 3D cell cultures (scale bar is 100 μm). (c) High magnification image of the same 3D cell culture as (b) with a scale bar of 20 μm. Adapted from “The relevance of using 3D cell cultures, in addition to 2D monolayer cultures, when evaluating breast cancer drug sensitivity and resistance,” by S. Breslin and O'Driscoll, 2016, Oncotarget, 7, pp. 45745–45756, with permission from Impact Journals.^28^ (d) 3D cell culture techniques and their advantages

Three‐dimensional cell culture technologies provide physiologically relevant and, likely, more predictive strategies for organogenesis^21^ and tissue engineering,^22^ organs‐on‐a‐chip,^23^ drug discovery and testing,^24^, ^25^ disease modeling,^26^ and developing cell‐based assays and animal‐free models.^27^ Three‐dimensional cellular systems, mimicking the native tissue structures, have been a noticeable improvement over two‐dimensional (2D) monolayer cultures in terms of improved cell–cell and cell–ECM interactions, high stability, and enhanced functionality (Figure 1).^29^, ^30^ Cell behavior and function are more realistic in 3D microenvironments, wherein an immense potential for predicting the efficacy of drug candidates similar to in vivo conditions may be found.^31^ An example of cells cultured in 2D and 3D systems are presented in Figure 1a–c, where a clear difference in morphology is observed for HER2‐overexpressing cell lines (HCC1954).^28^ The cells aggregated and formed tightly packed spheroids in the 3D cell culture, which is similar to their behavior in vivo.

To control the structure, morphology, and function of 3D cellular models, several strategies have been developed, including rotary cell cultures,^32^ microcarrier beads,^33^ gyratory shakers and roller tubes,^34^ spinner flask cultures,^35^ hanging drop method,^36^ liquid overlay cultures,^37^ and spontaneous cell aggregation methods (Figure 1d).^38^ Another approach is to encapsulate or seed cells in/on biomaterial scaffolds, providing a controllable microenvironment for the cells.

Here, we describe the physicochemical properties of gelatin and polysaccharides as natural biomaterials. We then focus on different combinations of gelatin and a variety of polysaccharides as scaffolds for 3D cell culture and tissue engineering. Merging these two building blocks may help design hybrid biomaterials that resemble the ECMs while providing additional therapeutic properties.

## 2 CHARACTERISTICS OF GELATIN AND POLYSACCHARIDES AS NATURAL BIOMATERIALS

**Figure 2:**
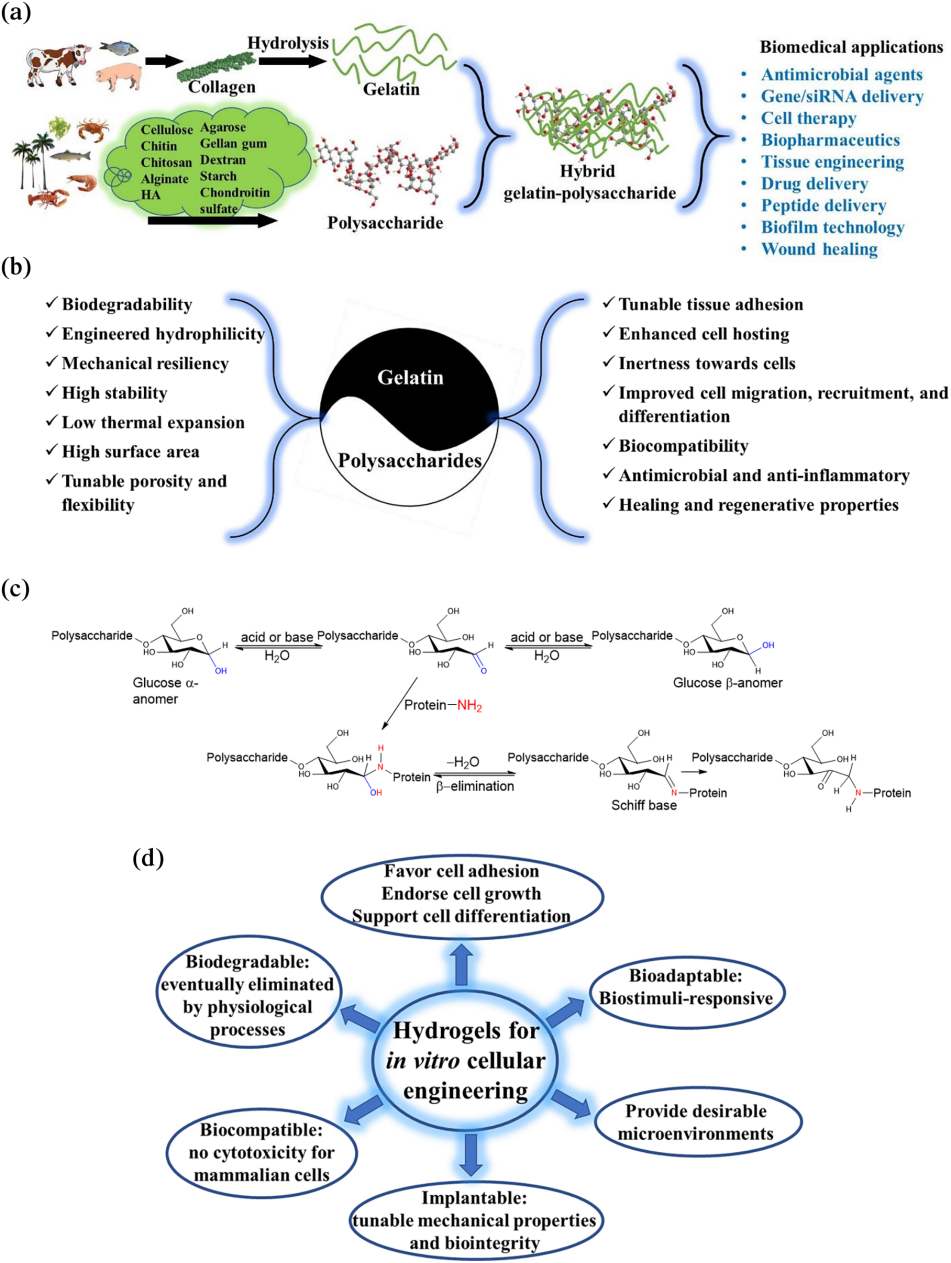
(a) An overview of the origin and significance of hydrogels prepared from gelatin and polysaccharides along with their biomedical applications. (b) Main characteristics of gelatin–polysaccharide scaffolds for 3D cellular engineering. (c) Chemical reactions between polysaccharides and proteins, encompassing Maillard reaction and Schiff base formation.^49^ In the first step, after an acid or base treatment, the polysaccharide ring opens, forming a reactive aldehyde moiety that further reacts with the primary amines of protein. After β‐elimination, the Schiff base adduct is formed, and further rearrangement yields stable products. (d) Desired properties of hydrogels for in vitro cell culture and tissue engineering

Gelatin and polysaccharides are natural biopolymers that have extensively been used for biomedical applications.^39^, ^40^ For example, our group has employed gelatin‐based materials, mainly gelatin methacryloyl (GelMA), in different biomedical applications, such as tissue engineering, bioprinting, and organs‐on‐a‐chip platforms.^41^, ^42^, ^43^, ^44^, ^45^, ^46^, ^47^, ^48^ Gelatin is a protein obtained from the hydrolysis of collagen, one of the main components of the ECM. As presented in Figure 2a, collagen may be derived from various sources, including bovine, porcine, or fish through various methods.^50^ Gelatin obtained from collagen via acid or base treatment is called type A or B, respectively.^51^ Different gelatin types acquire different characteristics, such as amino acid composition, gel strength (Bloom), isoelectric point (pI), and charge. For example, gelatin type A has a higher gel strength and glycine and proline contents. The pI for type A is between 8 and 9, exhibiting a positive charge at neutral pH; whereas, type B has a pI value between 4.8 and 5.4, bearing negative charge at neutral pH.^52^, ^53^

Polysaccharides may be sourced from crabs, lobsters, shrimps, forests (biomass), and bacteria (Figure 2a).^54^ In this review, polysaccharides such as cellulose, chitin, chitosan, alginate, and HA are highlighted and their synergy with gelatin in 3D cellular engineering is presented. The synergistic combination of gelatin and polysaccharides may result in improved properties, as presented in Figure 2b.

The interactions between carbohydrates and proteins may be engineered via two main chemical reactions leading to covalent bonding, resembling the proteoglycans in the ECM.^4^ These prominent reactions are Schiff base formation^55^ and Maillard reaction,^49^, ^54^ leading to hydrogel formation (Figure 2c). These reactions may explain why some ingredients or foods, such as bread, change color to brown as a result of carbohydrates–proteins interactions, setting a platform for efficient food quality control.^56^ Moreover, through this understanding, the formation of toxic products, such as heterocyclic amines and acrylamide, as well as taste variation in foods have been discovered.^57^ Recent studies have revolved around using gelatin and polysaccharides in biomedical applications, spanning from wound healing^58^ and cell growth^59^ to the inhibition of bacterial growth^60^ and the delivery of drugs, genes, siRNA, and peptides (Figure 2a).^61^

Gelatin–polysaccharide hydrogels may absorb a large amount of water, typically more than 100 times their dry mass, providing in vitro culture platforms to explore the behavior of mammalian cells in a matrix‐inspired environment for tissue engineering, favoring cell adhesion and growth, infiltration, and tissue vascularization (Figure 2d).^62^, ^63^ Polysaccharides typically increase the stability of scaffolds, and gelatin enhances the biological performance. Polysaccharides with various molecular weights, structures (e.g., linear or branched), functionality (monofunctional, containing only one type of functional group, for example, hydroxyl groups, or polyfunctional, bearing hydroxyl, carboxyl, and amino groups), and water affinity and solubility expand the library of ECM‐mimicking hybrid hydrogels. Furthermore, they induce gel formation upon mixing with proteins through a broad range of chemical and physical interactions, including electrostatic, hydrophobic, and hydrogen bonding. Additionally, certain applications of these two classes of biopolymers, for example, as toppings, are generally recognized as safe (GRAS), which may accelerate their translation from bench to bedside. The gelatin–polysaccharide composites can be prepared by a plethora of approaches, such as electrospinning, film casting, dip coating, physical mixing, layer‐by‐layer assembly, ionotropic gel formation, colloidal assembly, co‐precipitation, in situ preparation, and covalent coupling.^64^

One of the challenges associated with mixing these two classes of biomaterials is phase separation, which can have chemical and/or structural origins. The mixture of biomaterials undergoes phase separation when the timescale of gel formation is larger than that of the phase separation. Favorable interactions between proteins and polysaccharides originated from attractive forces may promote complex coacervation (association), and repulsive forces may lead to incompatibility (segregation).^65^, ^66^ Some crystalline polysaccharides, for example, certain cellulose, chitin, and chitosan often experience poor water solubility and phase separate upon mixing with gelatin.^67^ To overcome the phase separation of these biomaterials, they have been chemically modified to increase the water solubility and enhance their compatibility. In this regard, cellulose can be chemically modified to yield polyelectrolytes, such as carboxymethyl cellulose (CMC) and methylcellulose (MC), which are water soluble.^68^ However, despite the improved solubility, phase separation may still occur,^69^ which can be controlled by tuning temperature, ionic strength, and pH.^70^

## 3 GELATIN–POLYSACCHARIDES COMPOSITES IN CELL CULTURE AND TISSUE REGENERATION

In this section, we review state‐of‐the‐art hybrid hydrogels based on gelatin and polysaccharides to provide green and natural platforms for therapeutic cellular engineering. The polysaccharides mainly include cellulose, chitin, chitosan, alginate, and HA. Important examples of the hybrid hydrogels are discussed in terms of synthesis, fabrication, and their applications in cell culture and tissue engineering.

### 3.1 Gelatin–cellulose

**Figure 3:**
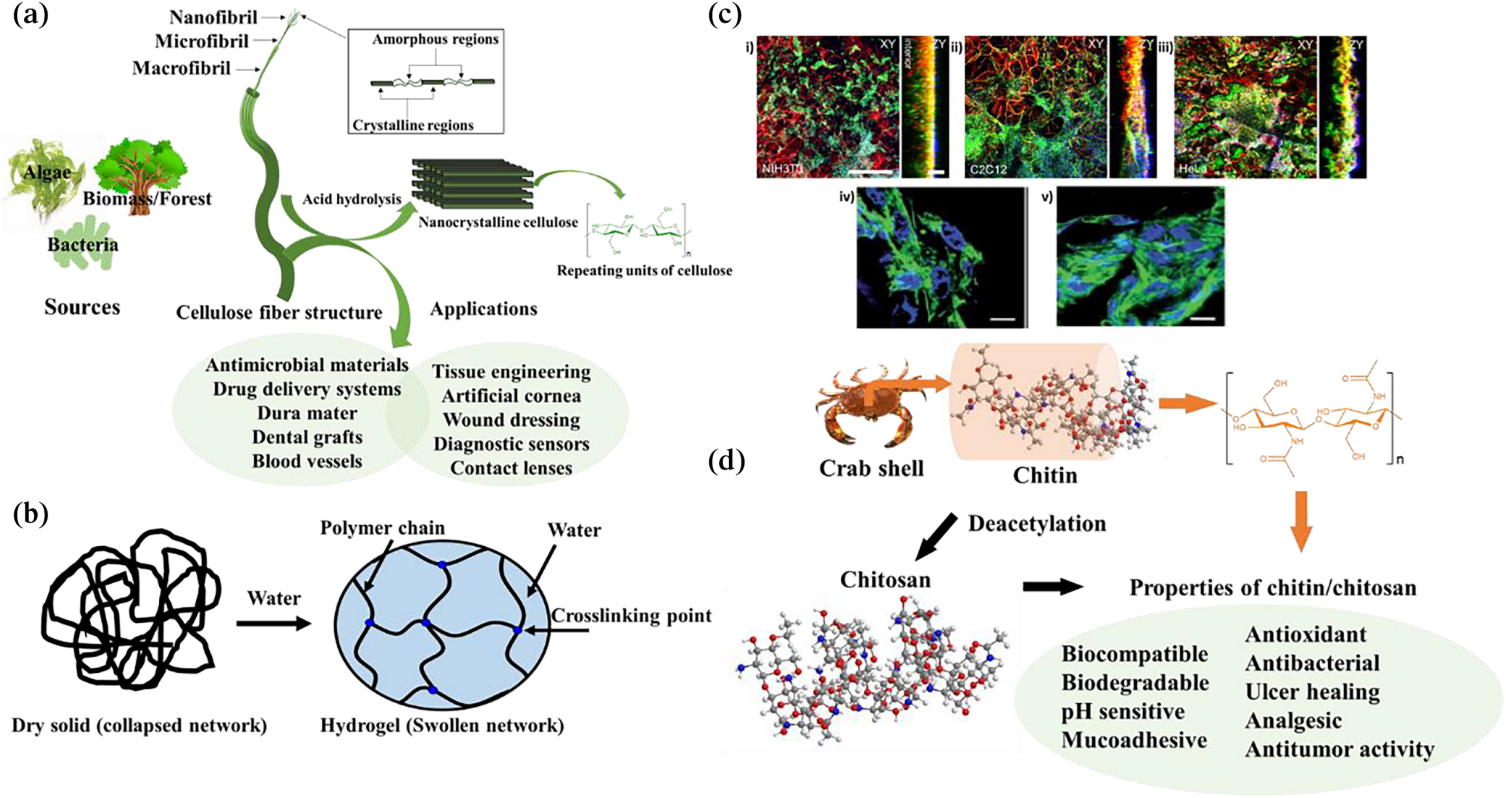
(a) Cellulose sources and classification based on size and structure; acid hydrolysis of the cellulose fibers, providing conventional cellulose nanocrystals; the representative structure of cellulose, comprising β(1→4) linked d‐glucose units, and its broad biomedical applications. (b) Illustration of dry anionic cellulose, which swells and forms a 3D hydrogel network in water. (c) Confocal microscopy images of stained cells, cultured in 3D cellulose scaffolds: (i) NIH/3T3, (ii) C2C12, and (iii) HeLa cells. Cellulose structure (red), mammalian cell membranes (green, stained with phalloidin conjugated to Alexa Fluor 488), and nuclei (blue, stained with DAPI). Adapted from “Apple derived cellulose scaffolds for 3D mammalian cell culture,” by D. J. Modulevsky et al., 2014, PLoS One, 9, p. e97835.^73^ Scale bars represent XY = 300 nm, ZY = 100 nm. (iv) DAPI/F‐actin merged images of stained NIH/3T3 cells after 7 days of incubation in a medium containing bacterial cellulose and (v) in microporous bacterial cellulose‐gelatin scaffolds. The scale bar represents 10 μm. Adapted from “Three‐dimensionally microporous and highly biocompatible bacterial cellulose–gelatin composite scaffolds for tissue engineering applications,” by S. Khan et al., 2016, RSC Adv, 6, pp. 110840–110849, with permission from Royal Society of Chemistry.^74^ (d) Chitin derived from the crab shell and its representative structure containing repeating units of disaccharide acetylglucosamine; *N*‐deacetylation of chitin results in chitosan, a polysaccharide made up of repeating units of randomly distributed β‐(1 → 4) linked d‐glucosamine and *N*‐acetyl‐d‐glucosamine; the properties of chitin and chitosan

Gelatin–cellulose scaffolds are less explored compared to other types of gelatin–polysaccharide hybrid biomaterials. Cellulose is the most abundant natural polymer on the earth, which benefits from properties, such as biocompatibility, renewability, biodegradability, cost‐effectiveness, hydrophilicity, and mechanical resilience.^71^, ^72^ Cellulose is a polysaccharide consisting of a linear chain of several hundreds to over 10,000 of β(1→4) linked d‐glucose units (Figure 3a).^75^, ^76^ Cellulose can be derived from the forest (biomass), algae, tunicate, and bacteria and be processed to form colloidal or fibrous materials, classified based on their size and morphology, which encompass macro‐, micro‐, and nano‐fibrillated or crystalline celluloses. The nano‐sized celluloses are mainly categorized as cellulose nanocrystals (CNCs), cellulose nanofibrils (CNFs), and bacterial nanocellulose (BNC).^77^, ^78^ CNCs are obtained after the acid hydrolysis of cellulose fibrils, wherein the amorphous regions are mostly hydrolyzed, yielding mainly the crystalline parts (Figure 3a).^79^, ^80^

The amorphous cellulose chains of fibrils may be oxidized using periodate and chlorite, yielding cellulose nanocrystals sandwiched between two highly functionalized protruding cellulose chains, resembling hairy cellulose nanocrystals.^81^, ^82^ Biologically instigated CNCs and CNFs, derived from renewable biomass, are currently receiving high attention due to their unique properties, such as high modulus (e.g., 100–200 GPa for CNC), low thermal expansion (3–22 ppm K^−1^ for CNC), and high surface area (400–500 m^2^ g^−1^).^83^, ^84^, ^85^, ^86^ In the context of 3D cellular engineering, high surface area of a scaffold may facilitate cell attachment, and high modulus provides stable and robust scaffolds for hard tissue/organ engineering.

Cellulose and its derivatives, such as cellulose acetate have been used for 3D cellular engineering.^87^ Recently, they have been used for the synthesis of cellulose nanoparticles, hydrogels, aerogels, and films for a wide range of biomedical applications, summarized in Figure 3a.^88^, ^89^, ^90^, ^91^ There are several approaches for designing cellulose‐based hydrogels, including the partial modification of hydroxyl groups by charged groups, promoting physical crosslinking.^92^, ^93^, ^94^, ^95^ Figure 3b illustrates the schematic of carboxylic acid‐modified cellulose in its dry form and the formation of water‐rich biopolymer networks after swelling. The content of carboxylic acid groups on the modified celluloses is vital for maintaining cell viability in the scaffolds. Carboxylated cellulose (oxidized cellulose) with 2.1 wt% of carboxylic groups showed a good compatibility with cells. Interestingly, low stability of the material at high acidity was observed (6.6 wt% of carboxylic groups), leading to disintegration and degradation in cell culture media.^96^ Different strategies can be adopted for designing carboxylated celluloses with a decreased acidity, yielding more compatible substrates for cells, e.g., through functionalization with arginine or the incorporation of chitosan to balance the acidity.^96^ High acidity prevents the adhesion of cells and influences cell growth, which may be caused by attracting free cations from cell culture media and increasing osmolality.^97^

Periodate‐oxidized cellulose nanocrystals may be mixed with gelatin to form a porous, 3D printable ink for fibroblasts.^98^ Furthermore, nanofibrillar cellulose has been combined with hyaluronan‐gelatin hydrogels for resembling the ECM.^99^ The composite biomaterial provided a scaffold for undifferentiated HepaRG cells, promoting the formation of spheroids with structural similarities to the liver tissue, such as functional bile canaliculi‐like structures and apicobasal polarity.

CNF‐based hydrogels for bone tissue engineering were doped with gelatin and β‐tricalcium phosphate as osteoconductive agents. The main role of CNF in these scaffolds was to decelerate degradation, inducing sustained release of an osteoinductive biomolecule (simvastatin). The scaffolds provided enhanced bone formation and better collagen matrix deposition compared to the control.^100^ Besides the biological benefits, cellulose nanofibers have imparted printability to gelatin‐based hydrogels, wherein CNFs enhanced the structural integrity and increased the mechanical stability of the composites.^101^ Bacterial cellulose–gelatin composite hydrogels have been used as versatile 3D scaffolds for culturing breast cancer cells to provide in vitro models of tumor microenvironments.^102^ Significant function of human breast cancer cell line (MDA‐MD‐231) in these scaffolds was reported.

The pore size and distribution play an important role in promoting cell proliferation, adhesion, and infiltration in scaffolds, where large pores permit nutrients to diffuse deep into the scaffolds while small pores promote cell differentiation and signaling.^103^ Infrared laser micromachining has been used to introduce macropores to bacterial cellulose scaffolds. Interestingly, a large number of pseudopodia were obtained with the scaffold, attesting to the strong adhesion of cancer cells to the scaffold, permitting multilayered cell formation.^104^ Biomimetic 3D cellulose sponge scaffolds may be prepared through electrospinning followed by sodium borohydride reduction to improve the mineralization capacity through nucleating calcium phosphate crystals. These sponges provide a temporary support for cell growth and migration,^105^ particularly in bone tissue regeneration, where biomimetic mineralization is essential. Laser‐patterned BC scaffolds modified with gelatin and hydroxyapatite have also been used for bone tissue engineering. These scaffolds were engineered to attain parallel pores, supporting the attachment, viability, and proliferation of chondrogenic rat cells.^106^ Chemical modifications, particularly TEMPO‐mediated oxidation, have been able to convert bacterial cellulose to a dispersant agent, enhancing the aqueous dispersion of hydroxyapatite nanoparticles. Adding gelatin to these dispersions, followed by crosslinking with glutaraldehyde provided a porous scaffold, supporting Calvarial osteoblasts for bone tissue engineering.^107^

Modulevsky et al. used apple‐derived cellulose for the 3D culturing of mammalian cells.^73^ The cellulose scaffolds were prepared by decellularizing apple hypanthium tissue (the edible part of an apple) using a detergent (sodium dodecyl sulfate) and used as 3D scaffolds for different cell types, such as NIH/3T3 fibroblasts, mouse C2C12 muscle myoblasts, and human HeLa epithelial cells. These mammalian cell types proliferated, migrated, and were viable for up to 12 weeks, wherein 98% of the cells remained viable in the culture (Figure 3c). In general, HeLa and C2C12 cells proliferated at higher rates than NIH/3T3 cells, and all the cells showed 3‐ to 4‐fold increase in number over the 12‐week culture.

Highly porous and 3D cell environments may be constructed using composite scaffolds comprising bacterial cellulose and gelatin.^74^ The porous nature of the composites favors water and nutrient infiltration into the scaffold, resulting in the improved growth and proliferation of cells. Bacterial cellulose and gelatin composite scaffolds have been fabricated using casting and particulate leaching approaches.^108^ The fabrication method permitted the preparation of porous scaffolds by dissolving the polymer in an organic solvent, followed by casting into a mold in the presence of porogen particles (e.g., salts). Subsequently, the solvent was evaporated, leaving the porogen‐containing scaffold. The polymer was then separated from the solid porogen at a high pressure, and after washing with water, the porous scaffold was yielded. The advantages of this method include the control of porosity; however, the drawbacks encompass limitations associated with mechanical properties and the incomplete removal of solvents and porogen additives.^109^ The incorporation of gelatin into bacterial cellulose‐based scaffolds resulted in improved biocompatibility, proliferation, and cell growth for NIH/3T3 fibroblasts (Figure 3c, iv,v).

Cellulose derivatives, such as CMC and MC, ethyl cellulose, acetyl cellulose, and hydroxypropyl cellulose have frequently been used to formulate hydrogels,^110^ nanoparticles (e.g., nanowhiskers),^111^ and nanofibers.^112^ Moreover, in combination with other synthetic and natural polymers, such as proteins,^113^ in particular gelatin,^114^, ^115^, ^116^, ^117^, ^118^, ^119^ a wide range of applications^120^, ^121^, ^122^, ^123^, ^124^, ^125^, ^126^ have been demonstrated for these composite materials as scaffolds for 3D cellular engineering.^127^, ^128^ Glycosaminoglycans (GAGs) in the ECM may be mimicked by electrospinning partially sulfated cellulose with gelatin, yielding functional fibrous structures. These scaffolds supported cell growth while electrostatically sequestered growth factors as a result of their charge originated from the spatial distribution of sulfate groups.^114^ Cellulose scaffolds containing the highest concentration of sulfate groups (5%) enhanced the mesenchymal stem cell (MSC) chondrogenesis, which was confirmed by a pronounced collagen type II production as a result of cartilage‐specific gene activation, attesting to the potential of partially‐sulfated cellulose in cartilage engineering.^129^

### 3.2 Gelatin–chitin

Chitin is an animal‐originated biopolymer, mostly obtained from invertebrates. It is available on the appendages as a structural component of arthropod animals in the cuticle region, e.g., exoskeleton of insects, spiders, and other crustaceans, namely crabs, lobsters, and shrimps.^130^, ^131^, ^132^ Chitin is a glucose derivative, homopolysaccharide made up of repeating chains of sugar molecules, explicitly N‐acetyl glucosamine moieties linked by a glycosidic bond (Figure 3d). Chitin has distinct biochemical properties that can regulate several biological activities, such as immune response and antibacterial actions. These properties have rendered chitin a favorable biomaterial in a wide range of applications, from scaffolds for 3D cell culture and tissue engineering^133^ to the treatment of medical conditions, such as inflammation,^134^, ^135^ and promoting wound healing.^136^, ^137^, ^138^, ^139^

Composite films of chitin nanofibers and gelatin have been prepared by casting and freeze‐drying highly viscous precursor solutions.^140^ The water content of chitin nanofiber‐gelatin biomaterials may be precisely engineered by tailoring the gelatin content.^140^ Increasing the gelatin concentration increased the swelling ratio. The nanofiber‐gelatin films did not induce inflammation and strongly promoted fibroblast proliferation, indicating high biocompatibility and bioactivity. In another work, chitin nanofibers‐GelMA nanocomposites were prepared via a self‐assembly approach, yielding ultra‐strong and flexible hydrogels.^141^ Compared to GelMA, the elastic modulus of these hydrogels was increased by ~1,000 folds, and the composite gel was 100 and 200% more extensible than chitin or GelMA, respectively. These hydrogels were used as scaffolds for human umbilical vein endothelial cells (HUVECs) cocultured with human mesenchymal stem cells (HMSCs), which provided enhanced cellular differentiation and vascular network formation due to the increased flexibility and elastic modulus.^141^

Embedding nanohydroxyapatite in β‐chitin–gelatin composites has enabled 3D cell culture for bone repair and regeneration.^142^ The cytocompatibility study of these scaffolds in mouse preosteoblast cells suggested that the cell behavior inside the microenvironment was regulated by the ions released from the hydroxyapatite particles. An increase in cell proliferation inside the nanocomposites resulted when phosphate and calcium ions were at their optimum concentrations, which would otherwise be toxic, leading to cell death. Gradual interfacial formation of calcium phosphate on chitin–gelatin membranes incubated in the simulated body fluid promoted facile attachment of human MG‐63 osteoblast‐like cells within 48 h. These cells reached full confluency on the bioactive membrane surface,^143^ which may provide implantable bone tissue engineering grafts.

α‐chitin and β‐chitin have been used to prepare regenerated and swelling hydrogels, respectively, in combination with gelatin and *N*‐acetyl‐d‐(+)‐glucosamine as a crosslinker at 120°C.^144^ While both types of hydrogels provided decent support for NIH/3T3 fibroblasts, the swelling ratio of β‐chitin‐based composites was higher than the regenerated hydrogels. Interestingly, the regenerated composite hydrogels underwent faster degradation than the swelling hydrogels.^144^ Accordingly, essential properties of gelatin hydrogels for tissue engineering, such as swelling, degradation, and mechanics may be readily tailored by tuning the chitin source and material processing method.

### 3.3 Gelatin–polycationic chitosan

Chitosan is a polycationic marine biopolymer obtained by *N*‐deacetylation of chitin, which has a broad spectrum of biological applications (Figure 3d).^145^ Important biological properties of chitosan are antitumor, antimicrobial, and antioxidant activities. The cationic nature of chitosan provides antibacterial properties and leverages electrostatic complex formation with negatively‐charged polymers.^146^ Nevertheless, the low solubility of chitosan in neutral or alkaline solutions is a major drawback, requiring further modification to improve its solubility. This can be improved by combining chitosan with gelatin either through the formation of a polyelectrolyte complex^147^ or by crosslinking.^148^

Chitosan–gelatin complexes exhibit structural similarities to both GAG and collagen in the ECM, providing favorable physicochemical and biological properties for cell culture. Hence, such complexes serve as a platform for tissue engineering and creating favorable environments for cell survival in vitro.^149^, ^150^, ^151^, ^152^ Chitosan imparts nonadhesiveness and a temperature‐tunable behavior to the complexes.^153^ Combining chitosan and gelatin at optimal ratios followed by crosslinking can tailor properties, such as mechanics, pore size, and cell viability.^154^ Chitosan and gelatin can be chemically conjugated to form hybrid biomaterials. Chemical crosslinking of chitosan and gelatin can be performed using 2,5‐dimethoxy‐2,5‐dihydrofuran (DHF), wherein DHF is activated with temperature in acidic media, forming dialdehyde groups that resemble the chemistry of glutaraldehyde crosslinking, followed by undergoing a Schiff base formation via the reaction with primary amines in chitosan and gelatin.^155^ Hybrid hydrogels prepared this way attain compressive moduli within the range of 0.284–1.167 MPa for uncrosslinked materials and 0.416–2.216 MPa for crosslinked ones, and pore size of ~220–260 μm (uncrosslinked chitosan–gelatin with volumetric ratio ~1) and 160–200 μm (crosslinked with volumetric ratio ~1 and crosslinking degree ~1). Tailoring gelatin content and crosslinking degree, the pore size, void space distribution, pore morphology, mechanics, and in vitro lysozyme‐mediated biodegradation have been engineered to support human keratinocyte cell (HaCaT) adhesion without any detectable genotoxicity.^154^

Miranda et al. employed a chitosan–gelatin composite as a scaffold for 3D bone marrow mesenchymal stem cell (BMMSC) culture.^156^ The porous biocomposite was prepared using glutaraldehyde crosslinking approach, which promoted cell adhesion, spreading, and viability. The scaffold showed good biocompatibility and slow degradation in vivo when implanted in the tooth sockets of a rat model. The implant stayed in place until the bone healing process was completed in 35 days.^156^ The crosslinked chitosan–gelatin composites benefit from interconnected pores, resulting in a decreased pore size compared to the uncrosslinked gel. The optimal gelatin concentration to obtain the highest cell viability (up to 90%) was about 25% beyond which (e.g., 50 and 100%) cell viability decreased (<40%). Importantly, the crosslinking procedure enhanced the cell viability as a result of the improved chemical stability, slow degradation, and the increased mechanical strength of composite scaffolds.

Gelatin concentration is a crucial parameter to tailor the mechanical stiffness of composite chitosan–gelatin biomaterials.^157^ For instance, the stiffness of hydrated chitosan (e.g., 1,660 kPa as a 2D substrate and 1.57 kPa as a 3D scaffold) and gelatin (90 kPa as a 2D substrate) was engineered by mixing them at a 1:3 chitosan:gelatin weight ratio, yielding 2D or 3D scaffolds with stiffness ~420 and 3.4 kPa, respectively. At a 3:1 chitosan:gelatin ratio, an increase in the stiffness for the 2D composite substrates (2,090 kPa) and a decrease for the 3D scaffold (1.15 kPa) were observed.^157^

**Figure 4:**
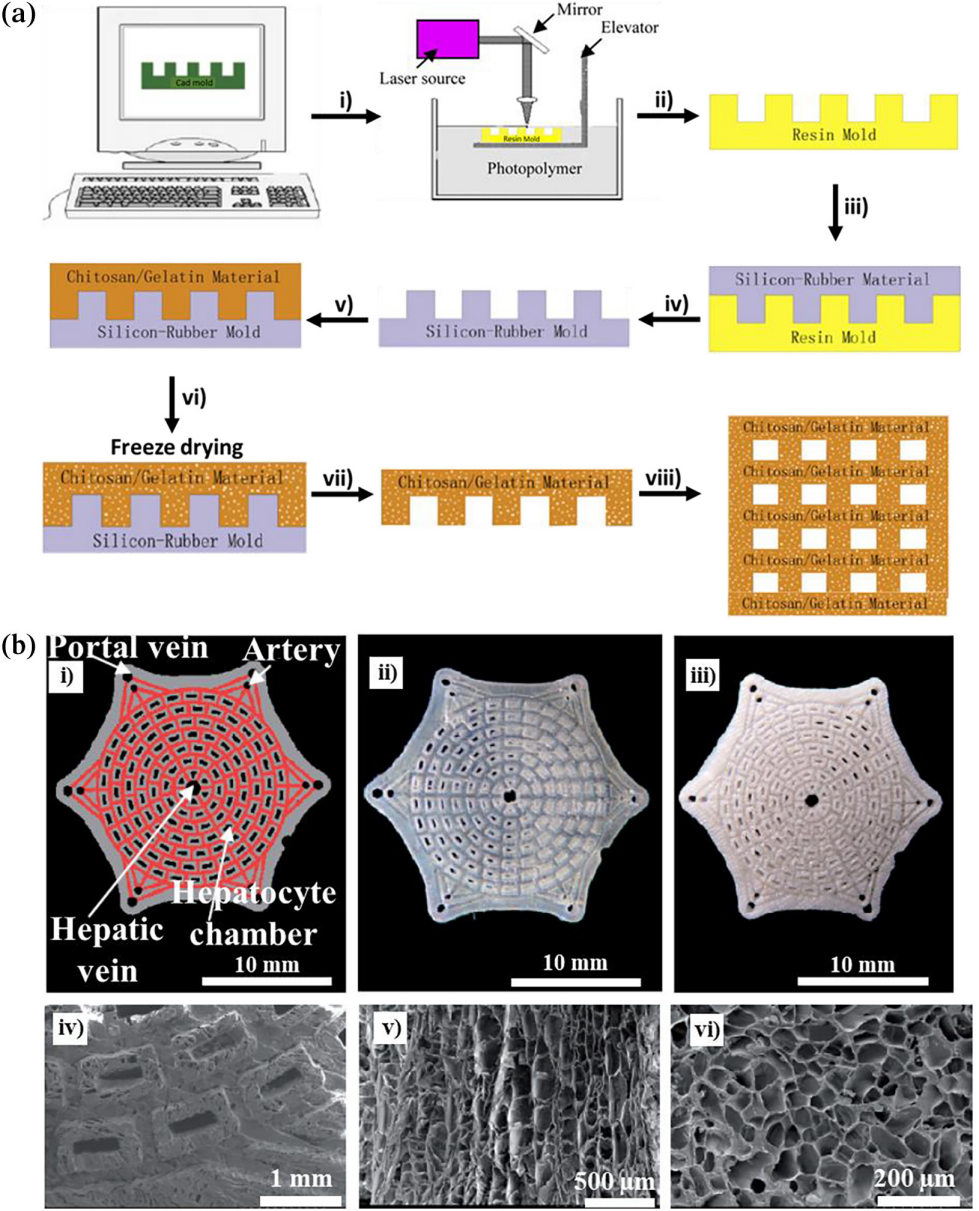
(a) Fabrication of chitosan‐gelatin scaffolds with well‐defined pore sizes. The designed model in CAD is used for the (i) preparation of resins by SFF technique, yielding (ii) the molds which are further used to prepare (iii) PDMS molds by the microreplication technique, followed by using (iv) the PDMS negative mold (v) to template the chitosan–gelatin solution, (vi) pre‐freeze‐drying the composite, (vii) drying the chitosan–gelatin scaffolds, and obtaining (viii) single‐layer scaffolds from which stacked scaffolds may be prepared. (b) Scaffolds with specific external shape and predefined internal morphology: (i) the CAD model, (ii) the resin mold, and (iii) the porous chitosan–gelatin scaffold. The SEM images of (iv) the predefined internal morphology and (v) the microstructure in longitudinal and (vi) transverse directions. Adapted from “Fabrication and characterization of chitosan/gelatin porous scaffolds with predefined internal microstructures,” by H. Jiankang et al., 2007, Polymer, 48, pp. 4578–4588, with permission from Elsevier^161^

The mechanical strength of gelatin–chitosan scaffolds can also be improved by the addition of β‐tricalcium phosphate, followed by freeze‐drying^158^ to yield porous scaffolds with interconnected pores for bone tissue engineering.^159^ Furthermore, microporous biomaterials based on chitosan and gelatin have provided promising scaffold platforms for the 3D culture of HepG2 cells.^160^ Large specific area with pore sizes ~100–200 μm have improved the viability, cell function, and proliferation. A well‐defined internal morphology of chitosan–gelatin scaffolds, wherein the microstructures were precisely controlled by micromanufacturing, mimicked the network configuration of hepatic chambers and portal and central veins. These engineered scaffolds promoted the hepatocyte cell function, characterized by large colony formation in the predefined chambers within 1 week, which secreted albumin and urea more effectively than highly porous materials.^161^, ^162^, ^163^ These scaffolds were prepared through the combination of solid freeform fabrication (SFF),^164^ microreplication,^165^ and freeze‐drying approaches.^166^ The fabrication process is described in Figure 4a. Initially, the desired shape was programmed using a computer‐aided design (CAD) software from which a resin mold, typically from polydimethylsiloxane (PDMS), was prepared. Subsequently, the chitosan–gelatin solutions were added to the patterned PDMS molds and freeze‐dried, providing a well‐defined porous scaffold. The 3D scaffolds were prepared by stacking single‐layer structures. This fabrication technology enabled the design of microenvironments comparable to the highly organized liver structure as presented in Figure 4b.

Other biopolymers have also been added to chitosan–gelatin composites to improve their functionality. For example, chondroitin sulfate was mixed with them to establish 3D porous scaffolds that support and enhance the differentiation of MSCs to osteoblasts for bone defect repair.^167^ The addition of HA and heparan sulfate to chitosan–gelatin promoted neural stem and progenitor cell adhesion, growth, and differentiation in 3D environments.^168^ Moreover, electrospun PCL, chitosan, and gelatin nanofibers with tunable mechanical properties have been used for skin tissue engineering.^169^, ^170^

The addition of glycerol phosphate to chitosan and gelatin resulted in a hydrogel with tunable gel formation time, which was used as a 3D scaffold for nucleus pulposus regeneration.^171^ For the application in bone tissue engineering, gelatin‐chitosan composites demonstrated a similar strength to natural bones with compressive strength ~2–12 MPa and Young's modulus ~50–500 MPa (for cancellous bone).^172^ These requirements were provided by the combination of chitosan‐gelatin composites with hydroxyapatite^173^, ^174^ or nano‐bioglass.^175^ The mechanical properties of these composites were significantly enhanced by the addition of bioglass (30%), yielding compressive strength ~2.2 MPa and elastic modulus ~111 MPa. In this example, the compressive and elastic moduli of gelatin were 0.8 and 5.23 MPa, respectively. The addition of hydroxyapatite was also able to increase the compressive strength (3.17 MPa) and Young's modulus (310 MPa) compared to the gelatin–chitosan composites (1.33 and 120 MPa, respectively).

### 3.4 Gelatin–crosslinkable alginate

**Figure 5:**
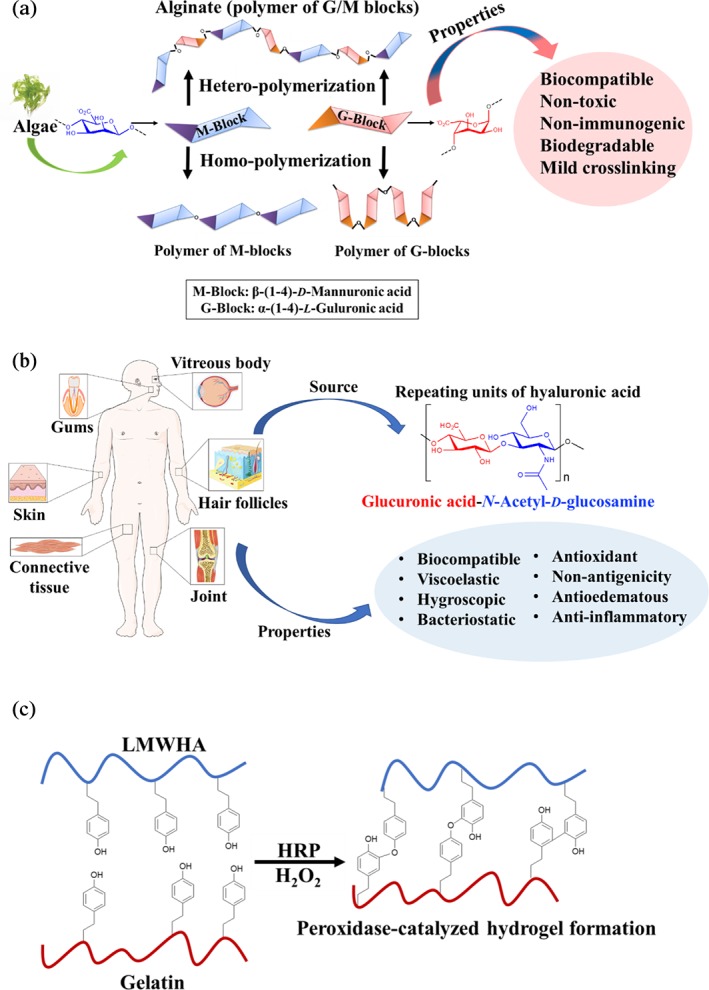
(a) The source of alginate and its representative structure composed of guluronic acid (G‐block) and mannuronic acid (M‐block) units, which may form three kinds of polymers in the presence of divalent ions, such as calcium. (b) The presence of HA in different parts of the body, its representative structure^180^ comprising glucuronic acid and *N*‐acetyl‐d‐glucosamine, and main properties. (c) Horseradish peroxidase (HRP)‐catalyzed hydrogel formation by reacting low molecular weight HA (LWHA) with gelatin^181^

Alginate is a biopolymer extracted from seaweeds, such as brown algae, *Ascophyllum, Durvillaea, Ecklonia, Laminaria, Lessonia, Macrocystis, and Sargassum spp*.^176^ Alginate is a polyelectrolyte with two molecular building blocks, which regulate its structural properties and promote its mild crosslinking in the presence of divalent cations, leading to the formation of strong and structured hydrogels.^177^, ^178^, ^179^ The building blocks of alginate are guluronic acid (G‐block) and mannuronic acid (M‐block), permitting the formation of a hydrogel in the presence of divalent cations, such as calcium (Ca^2+^), which act as a physical crosslinker (Figure 5a).^182^

Alginate properties include biocompatibility, nontoxicity, nonimmunogenicity, and biodegradability.^183^, ^184^ These desirable properties support the development of biomaterials for tissue engineering with therapeutic values.^185^ Properties of alginate hydrogels can be tailored through the modification of free hydroxyl and carboxyl groups to regulate solubility, hydrophobicity, and biological characteristics pertinent to cell adhesion and survival.^186^, ^187^ Several efforts have been devoted to develop chemical strategies for the modification of alginate, encompassing oxidation, sulfurylation, esterification, and amidation to impart additional properties to the biopolymer.^188^ For example, the anticoagulant, anti‐inflammatory, and antitumor activities of alginate can be engineered through sulfurylation.^189^, ^190^ The alginate degradation can be improved by partial oxidation,^191^ and through esterification, a more hydrophobic biomaterial with improved gel strength can be prepared.^192^

Furthermore, alginate has widely been used as a hydrogel for the construction of artificial 3D ECM and models for drug testing.^193^, ^194^, ^195^ Optimal concentration and viscosity of alginate hydrogels are fundamental for developing a suitable cell culture model. For example, alginate hydrogels were used as scaffolds for Hepatic Huh‐7‐cell line, providing tissue models for the in vitro study of Hepatitis C virus infection.^196^ Low viscosity alginate (e.g., 200 mPa s, 1%) yielded a material with low stability, whereas the medium viscosity alginate (2,000 mPa s, 2%) resulted in a stiff material, which prevented the cell proliferation. Decreasing the alginate concentration to 1.5% provided an optimal microenvironment for the cells, reflected in the albumin production and CYP1A activity.

Chemical composition of alginate hydrogels, regulated by the ratio of the G‐block to M‐block, is another important factor that impacts the mechanical properties. Dominant G‐block (G‐type alginate, 1.2%) yielded a rigid and elastic gel with viscosity η = 262 mPa s and storage modulus *G*′ ~ 31.1 kPa, while the linear M‐block (M‐type alginate, 1.2%) provided a more viscous and less elastic material (η = 440 mPa s, *G′* ~ 9.9 kPa, Figure 5a).^197^ The M‐type gels have been suitable for cardiac patches and the gels with dominant G‐block are promising candidates for cardiac implants.^197^ Cell‐laden hydrogels made up of alginate and gelatin have numerous advantages, including controlled pore size and distribution as well as cell protection against external physical and chemical stimuli.^198^, ^199^ Alginate is a nonporous biomaterial; therefore, the porosity of composite alginate–gelatin hydrogels can be controlled by tuning the gelatin content.^200^ The porosity of the composites may be engineered through the addition of gelatin beads with various sizes (150–300 μm) physically crosslinked at low temperature (4°C), followed by heat‐mediated dissolution inside alginate scaffolds.^200^ These hydrogels benefited from 2 to 3 orders of magnitude increased permeability; however, their compression modulus decreased.

Recently, 3D printing technology has received attention in therapeutic and clinical applications.^201^ Capability to construct personalized 3D structures introduces a wide range of possibilities to address clinical challenges, such as the design of optimal prosthetics or implants compatible with the host tissue. In this context, the choice of proper biomaterial combinations that resemble the ECM structure and permit the manufacturing of cell‐laden constructs is vital.^202^, ^203^ Recent 3D bioprinting technologies can help generate engineered blood vessels,^204^ artificial skin,^205^ cartilage,^206^ and a wide range of tissue constructs.^207^ The combination of gelatin and alginate has provided a platform to preserve cell function and survival within printed constructs, promoting the repair of lesions.^208^

Alginate–gelatin bioinks have recently stimulated the field of 3D printing^209^, ^210^ and bioprinting, leveraging robust, cell‐friendly, and facile fabrication of cell‐laden hydrogel constructs.^211^, ^212^ Alginate–gelatin composites, wherein gelatin functions as a stabilizer, have been used for the 3D bioprinting of osteosarcoma (Saos‐2) cell‐laden scaffolds; however, the printed scaffolds did not promote cell proliferation.^213^ Nevertheless, incubating the printed constructs with agarose and calcium polyphosphate enhanced the cell proliferation and increased the Young's modulus from 13–14 kPa to 22 kPa. Bone morphogenetic protein‐2 (BMP‐2)‐loaded gelatin microparticles were embedded in bioprinted alginate to induce osteogenicity in rodent (mice and rats) models.^214^ The bioink included biphasic calcium phosphate and goat multipotent stromal cells (gMSCs), which provided sustained BMP‐2 release for 3 weeks, promoting osteogenic differentiation and bone formation.

Degradation rate of alginate‐based bioprinted scaffolds can be tailored by tuning the ratio of sodium citrate to sodium alginate. Human corneal epithelial cells (HCECs) were bioprinted in collagen–gelatin–alginate composite hydrogels, and the scaffolds were exposed to sodium citrate, yielding controlled degradation, which in turn resulted in high cell viability (>90%), proliferation, and cytokeratin 3 (CK3) expression.^215^ Alginate–gelatin bioinks can also be engineered by tailoring the ionic strength.^216^ The storage and loss moduli of bioprinted constructs decreased using 1× (165 mM) and 2× (328 mM) phosphate‐buffered saline (PBS), resulting in mechanically weak, fast‐swelling, and unstable scaffolds, incapable of hosting epidermal stem cells. Similarly, without PBS, the cells remained isolated from each other and were not able to proliferate. The optimum concentration of PBS (82 mM, 0.5×) resulted in improved cell function in terms of viability, proliferation, glandular morphology, and differentiation to epithelium and sweat glands, while providing a decent printability of epidermal stem cell‐laden constructs, setting the stage for the regeneration of sweat glands.^216^

Developing clinically relevant models of tumors has been a prime impetus for emerging 3D culture systems.^217^, ^218^ A bioink consisting of gelatin, alginate, and fibrinogen hydrogels combined with HeLa cells was used to 3D print cervical tumor models and investigate disease pathogenesis and drug resistance.^219^ In the 3D bioprinted model, HeLa cells expressed high levels of matrix metalloproteinases (MMPs) and high chemoresistance, resembling an in vivo tumor. These composite hydrogels overcome the poor degradation of printed cell‐laden alginate constructs, which would otherwise negatively impact cell proliferation. Metabolic activity of tumors under chemotherapy has been modeled using alginate‐based cancer cell‐laden 3D scaffolds. Encapsulated human hepatoma (HepG2) liver cells in alginate hydrogels were exposed to a coumarin pro‐drug, resembling the in vivo drug metabolism.^220^ These models have helped minimize the necessity of animal models and may better reflect the outcome in human trials.

### 3.5 Gelatin–hyaluronic acid

Hyaluronic acid is a GAG, an ECM component in many parts of the body, such as vitreous body,^221^, ^222^ gums,^223^ connective tissue,^224^ skin,^225^ and joint,^226^ which promotes cell motility and connects tissues. Due to its abundance in the body, it is used as a suitable biomaterial to treat wounds^227^, ^228^ and medical conditions such as hypertension,^229^ bone defects,^230^ osteoarthritis,^231^ and neurological disorders.^232^ Furthermore, HA plays a key role in developing tissue culture scaffolds^180^ and cosmetic materials.^233^ Originally, HA was discovered in the vitreous humor of the eye and realized to be made up of two monomers, namely glucuronic acid and *N*‐acetyl‐d‐glucosamine polymerized into large macromolecules of over 30,000 repeating units (Figure 5b).^234^

Features of HA, such as biocompatibility, hygroscopicity, viscoelasticity,^235^ bacteriostatic and antioxidant effects,^236^ nonantigenicity,^237^ antiedematous^238^ and anti‐inflammatory properties^239^ render it extremely attractive in various therapeutic technologies for body repair.^240^, ^241^ The combination of HA and gelatin has been employed as a semi‐permanent dermal filler, wherein HA provided the structural integrity, and gelatin promoted host tissues integration.^242^ The two components were crosslinked using 1‐ethyl‐3‐(3‐dimethylaminopropyl)carbodiimide.^243^ The in vivo experiments were performed by subcutaneously injecting the gel in the back of rats, resulting in tissue ingrowth after 4 weeks, which indicated that the material promoted cell infiltration and new tissue formation with no cytotoxicity.

Gelatin–HA‐based biomaterials have also been used for wound dressing. To this end, it is important that the material provides a warm and moist environment to facilitate wound healing.^244^ Optimal moisture condition (2,000–2,500 g m^−2^ day^−1^)^245^ was targeted by altering the composition of these gels (gelatin:HA ~ 8:2, 5:5, and 2:8 wt%:wt%). It has been demonstrated that 8:2 gelatin:HA provided the fastest wound healing in vivo (95% wound healing on day 10 in a mouse full‐thickness wound model compared to 78% for the control) with an optimal water vapor transmission rate ~2,670 g m^−2^ day^−1^.

Several chemical modifications have been performed on HA to tailor its properties and facilitate crosslinking for hydrogel formation.^246^ A common modification strategy is to functionalize HA with thiol groups, yielding a biocompatible hydrogel with therapeutic properties. Thiolated HA (3,3‐dithiobis‐[propanoic dihydrazide]) or thiol‐carboxymethyl HA can be crosslinked to form a hydrogel by the addition of PEG diacrylate. These composites have been used as injectable scar‐free 3D cell scaffolds for wound healing or as 3D cell culture scaffolds.^247^, ^248^, ^249^ Cyto‐adhesiveness of the material can be increased by co‐crosslinking with gelatin, modified with thiol groups, yielding a gel with tripeptide Arg‐Gly‐Asp (RGD) motifs for binding integrins on cell surfaces.^250^ Moreover, HA can be chemically modified to acquire hydrophobic properties, which may be homogeneously mixed with gelatin to form hydrophobic–hydrophilic mixed gels used as 3D scaffolds for the chondrogenic differentiation of MSCs.^251^ An optimal balance of hydrophobic and hydrophilic properties of these hydrogels may have a noticeable impact on their performance.^252^ The hydrophobic nature of the material plays an important role in the mechanical strength, and additionally, allows the cells to adhere to the material surface rather than infiltrating within. These hydrogels, however, inhibit cell encapsulation by impairing the diffusion of water, nutrients, and wastes to and from cells.^253^

Combining low molecular weight HA and gelatin through the peroxidase‐catalyzed hydrogel formation was examined to encapsulate endothelial cells.^181^ Initially, HA and gelatin were covalently functionalized with 4‐hydroxyphenyl groups for the enzymatic crosslinking and hydrogel formation (Figure 5c). These 3D biomaterial scaffolds had high compatibility and motility for HUVECs. In another work, Singh et al. designed a 3D macroporous material based on HA, gelatin, and alginate, which was crosslinked using calcium chloride.^254^ The component selection was based on the unique properties that each biopolymers provided: gelatin was chosen to promote cell adhesion and cell–cell interactions, alginate to provide good encapsulation properties and inertness towards cells, and HA to enhance stem cell migration and differentiation and promote osteogenesis. The composite hydrogel promoted the osteogenic differentiation of stem cells for bone tissue engineering in vivo. The scaffold had the ability to recruit cells, prominently promoting effective integration with the host tissue within 1 week without any significant immune reaction.

HA has also been combined with GelMA, providing a robust and decent candidate for 3D cell culture due to its ability to form a composite network after a mild photocrosslinking.^41^ The combination of GelMA and methacrylated HA improved the mechanical properties of the composite hydrogels.^255^, ^256^, ^257^ Furthermore, the physical and biological properties of the combined gels were tunable via changing the composition. Interestingly, in the absence of GelMA, the HUVECs did not show any spreading in the 3D hydrogels, highlighting the importance of the synergistic action in polysaccharide–gelatin biomaterials.

### 3.6 Gelatin combined with other polysaccharides

Polysaccharides have proven to be good supplementary biomaterials for gelatin, improving its applicability and properties (Figure 2b).^258^, ^259^ Besides the polysaccharides discussed so far, which have widely been combined with gelatin, in this section, less‐explored polysaccharides for 3D cellular engineering and therapeutic applications will be highlighted. Table 1 presents these polysaccharides with their structure and applications.^260^, ^261^, ^262^, ^263^, ^264^, ^265^, ^266^, ^267^, ^268^, ^269^

**Table 1:** Less‐explored polysaccharides that have been used in combination with gelatin‐based biomaterials, their structures, and applications

Polysaccharide	Chemical structure	Applications
Agarose	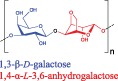	Separation of biomolecules Scaffolds for tissue engineering Carriers for drug delivery
Gellan gum	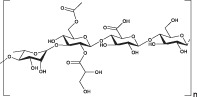	Drug delivery vehicles Injectable hydrogels Cell delivery materials
Dextran	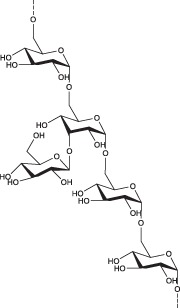	Tissue adhesive materials Drug delivery Tissue repair
Starch	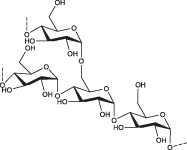	Application in food industry Plastic polymer production Filler material as nanoparticles
Chondroitin sulfate	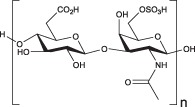	Tissue engineering Osteoarthritis management Anti‐inflammatory agents Wound healing

Here, we provide examples wherein gelatin and agarose have been merged for 3D cellular engineering.^270^ Agarose is a ubiquitous polysaccharide obtained from agar.^271^ Different concentrations of agarose with gelatin (agarose:gelatin ~ 100:0, 75:25, 50:50, and 25:75 wt%:wt%) have been investigated for tuning chemical, mechanical, and biological properties. The samples with 50 wt% agarose formed gels at the body temperature and exhibited high stability and mechanical resilience, in which case ~95% of the gels stayed intact and maintained their shape. The stability of the gels was evaluated by the shear force rupture assay.^272^ This concentration of agarose provided the best cell attachment and a decent structural integrity. Interestingly, increasing the agarose concentration to 100 wt% resulted in a weak mechanical stability due to the formation of a more fragile gel (only 80% of the gel remained intact). Bhat and Kumar used agarose in combination with chitosan and gelatin to form a cryogel as a potential 3D scaffold for skin and cardiac tissue engineering.^273^ The cryogel promoted cardiac and fibroblast cell growth and proliferation; however, the cells underwent fast initial proliferation within 24 hr due to the rapid contact with the matrix in 2D cell culture compared to the 3D scaffold. After 3 days, the 2D system induced cell death as a result of limited nutrients and interfacial attachment sites; whereas, in the 3D scaffold, despite a slow initial proliferation as a result of large surface area (demanding more time to establish cell–cell interactions), the cells proliferated for a longer period. Once adhered to the 3D scaffold, the cells had more space to proliferate and benefited from large pore sizes that facilitated the diffusion of oxygen and nutrients and prolonged proliferation, which was otherwise implausible in the 2D cultures.

Gellan gum, a polysaccharide produced by microbial fermentation of *Sphingomonas paucimobilis* microorganism,^274^, ^275^ has been combined with GelMA as a bioink for 3D cartilage bioprinting.^276^ The addition of gellan gum had a significant impact on the printability of the material by increasing the yield stress (0.13 Pa for 10 wt% GelMA and 48.2 Pa for GelMA:gellan gum ~ 10:0.5 wt%:wt%) and stiffness (Young's modulus ~ 24.1 kPa for 10 wt% GelMA and 77.8 kPa for GelMA:gellan gum ~ 10:1 wt%:wt%). The printed constructs promoted the generation of a support matrix by scaffold‐embedded chondrocytes. High concentrations (≥9 wt%) of gellan gum led to the formation of a rigid solid, hampering cell encapsulation, and on the contrary, low concentrations (0.20 wt% gellan gum and 15–20 wt% GelMA) resulted in a liquid‐like, unprintable material. The optimal concentration for decent printability and cartilage tissue formations was ~10 wt% GelMA and 0.5 wt% gellan gum.

Dextran in combination with gelatin can provide a suitable 3D scaffold with potential applications in 3D cellular engineering.^277^ In order to prepare a composite hydrogel, dextran and gelatin can be separately modified to undergo crosslinking. To this end, two main approaches have been reported: (a) dextran was oxidized to its corresponding dialdehyde using sodium periodate, and in parallel, gelatin was modified by a reaction with ethylenediamine to increase amino groups. These biomaterials rapidly formed a Schiff base upon mixing, providing a hydrogel without requiring any catalyst^278^; (b) dextran was modified with methacrylate groups and lysin, and gelatin was methacrylated (GelMA), providing a UV light crosslinkable pregel solution.^279^ The mechanical properties of these hydrogels were controlled by tuning the degree of functionalization, yielding hydrogels with storage moduli ~ 900–6,100 Pa. The designed hydrogels were used as 3D scaffolds for synovium‐derived MSCs, promoting their differentiation into chondrocytes, which were injected subcutaneously in nude mice.^280^ The in vivo experiments showed that the cell‐laden hydrogels promoted the formation of new cartilage after 8 weeks without any significant evidence of inflammation.

Another interesting polysaccharide that has been studied in combination with gelatin for cellular engineering is starch. This combination has been a promising scaffold for promoting the adhesion and proliferation of adipose tissue‐derived stem cells due to a similar chemical structure to the ECM.^281^, ^282^ However, it is necessary to optimize the biomaterial composition because starch can cause cell detachment. Therefore, a wide range of concentrations (gelatin: starch ~20–58 wt%:wt%) was evaluated, and at low gelatin concentrations, partial cell detachment was observed.

Chondroitin sulfate, one of the major components of cartilage ECM, has important therapeutic properties, such as anti‐inflammatory effects while promoting wound healing by increasing cellular adhesion and proliferation during the healing process.^283^ To benefit from these properties, chondroitin sulfate has been combined with gelatin and HA for developing 3D scaffolds for cartilage tissue engineering^284^ and skin substitutes.^285^ Addition of chondroitin sulfate to gelatin improved the resistance against collagenase‐induced degradation, preserving the storage modulus and porosity of gelatin, while HA promoted the integration of engineered cartilage with the host tissue and improved the scaffold strength.

## 4 CHALLENGES AND FUTURE DIRECTIONS

There remain some limitations and challenges to overcome in order to devise ideal biomaterials for advanced 3D cell culture and tissue engineering applications based on the composites of gelatin and polysaccharides. Design of hybrid biomaterials with desired physical, chemical, and biological properties at physiological conditions based on facile preparation and sterilization technologies requires precise and scalable manufacturing processes. Moreover, developing hybrid biomaterials with tunable degradation in biological environments is pivotal for biomedical applications. While gelatin is readily biodegraded in vivo by several enzymes, such as collagenase, polysaccharide degradation may be more challenging. For instance, cellulose, alginate, and agarose cannot be enzymatically degraded in the human body due to the lack of cellulose‐degrading enzyme cellulase, alginate‐degrading enzymes alginate lyases,^286^ and agarose‐degrading enzyme agarase; however, several strategies can be adopted to promote the in vivo biodegradation of polysaccharides. For example, through chemical modifications, such as the oxidation of regenerated cellulose,^287^ and the incorporation of relevant enzymes into the scaffold have facilitated the degradation of polysaccharides.^288^, ^289^ For alginate and agarose, similar strategies can be employed. Interestingly, it has been demonstrated that these polysaccharides can be degraded (fermented) in the gastric intestinal tract by gut microbiota.^290^, ^291^ Other polysaccharides can also be degraded by enzymes^292^: chitosan or chitin (using lysozyme), hyaluronic acid (hyaluronidase, β‐d‐glucuronidase and β‐*N*‐acetyl‐d‐hexosaminidase), starch (α‐amylase), chondroitin sulfate (β‐glucuronidase, β‐*N*‐acetylgalactosaminidase and chondroitinase),^269^, ^293^ dextran,^294^ and guar gum (degradable by the enzymes produced by a bacterium in the human colon).^295^

Even though polysaccharides are typically biocompatible and nontoxic, cares must be taken to thoroughly understand the biocompatibility of their degradation byproducts. For example, the production of immunogenic substances during the degradation of cellulose^296^ must carefully be assessed in vivo before translating the hybrid hydrogels for clinical applications. Future research should endeavor to expand the applications of gelatin–polysaccharide hybrid biomaterials for mimicking the role, associated molecular pathways, and chemistry of glycoproteins in the ECM.

## 5 CONCLUSIONS

Natural biomaterials have leveraged the cell behavior and function, enabling the advancement of tissue engineering for therapeutic applications. To better mimic the physiological, biochemical, and physical cues of native tissues, natural hybrid 3D scaffolds have extensively been explored. The success of 3D cellular structures is contingent on the development of functional biomaterials that are endowed to heal, repair, or regenerate injured or diseased tissues and organs. We have reviewed hybrid gelatin–polysaccharide biomaterials as naturally derived therapeutic scaffolds that can overcome some of the limitations of synthetic polymeric materials and mimic the building blocks of ECMs. Polysaccharides, such as cellulose, chitosan, chitin, HA, and alginate with their superior properties complement the missing capabilities of gelatin. These composite biomaterials may leverage the field of therapeutics by providing cues that would otherwise be impossible to obtain from the individual components. We believe that the synergistic potentials of this class of composites will pave the way for developing superior precision therapeutics based on natural and cost‐effective biomaterials with well‐defined characteristics.
